# Genome-wide analysis of the *CCT* gene family in Chinese white pear (*Pyrus bretschneideri* Rehd.) and characterization of *PbPRR2* in response to varying light signals

**DOI:** 10.1186/s12870-022-03476-1

**Published:** 2022-02-23

**Authors:** Zheng Liu, Jia-Li Liu, Lin An, Tao Wu, Li Yang, Yin-Sheng Cheng, Xian-Shuang Nie, Zhong-Qi Qin

**Affiliations:** 1 Research Institute of Fruit and Tea, Hubei Academy of Agricultural Sciences, Wuhan, 430064 China; 2 College of Life Sciences, Wuhan University, Wuhan, 430072 China; 3 Key Laboratory of Horticultural Plant Biology (Ministry of Education), Huazhong Agricultural University, Wuhan, 430070 China

**Keywords:** Pear, CCT family, Phylogenetic analysis, Expression profile, Light environment, Transient overexpression

## Abstract

**Background:**

Canopy architecture is critical in determining the light environment and subsequently the photosynthetic productivity of fruit crops. Numerous CCT domain-containing genes are crucial for plant adaptive responses to diverse environmental cues. Two *CCT* genes, the orthologues of *AtPRR5* in pear, have been reported to be strongly correlated with photosynthetic performance under distinct canopy microclimates. However, knowledge concerning the specific expression patterns and roles of pear CCT family genes (*PbCCTs*) remains very limited. The key roles played by *PbCCTs* in the light response led us to examine this large gene family in more detail.

**Results:**

Genome-wide sequence analysis identified 42 putative *PbCCTs* in the genome of pear (*Pyrus bretschneideri* Rehd.). Phylogenetic analysis indicated that these genes were divided into five subfamilies, namely, COL (14 members), PRR (8 members), ZIM (6 members), TCR1 (6 members) and ASML2 (8 members). Analysis of exon–intron structures and conserved domains provided support for the classification. Genome duplication analysis indicated that whole-genome duplication/segmental duplication events played a crucial role in the expansion of the CCT family in pear and that the CCT family evolved under the effect of purifying selection. Expression profiles exhibited diverse expression patterns of *PbCCTs* in various tissues and in response to varying light signals. Additionally, transient overexpression of *PbPRR2* in tobacco leaves resulted in inhibition of photosynthetic performance, suggesting its possible involvement in the repression of photosynthesis.

**Conclusions:**

This study provides a comprehensive analysis of the *CCT* gene family in pear and will facilitate further functional investigations of *PbCCTs* to uncover their biological roles in the light response.

**Supplementary Information:**

The online version contains supplementary material available at 10.1186/s12870-022-03476-1.

## Background

CCT [CONSTANS (CO), CONSTANS-LIKE (COL) and TIMING OF CAB1 (TOC1)] transcription factors, which encode proteins with a conserved motif (CCT domain) of ~ 43 amino acid residues towards their carboxy-terminus, constitute a plant-specific family [1, 2]. The CCT domain has important functions in nuclear localization, protein–protein interactions, transcriptional regulation and nuclear protein transport [3–5]. CCT family genes can be divided into five categories based on the sequencing information of *Arabidopsis*: COL, PRR (Pseudo-Response Regulator), ZIM (Zinc-finger Protein Expressed in Inflorescence Meristem), TCR1 (Tunicamycin-induced COL-Related 1) and ASML2 (Activator of Spo^min^::LUC2) subfamilies [6, 7]. The CCT family has been comprehensively analysed in some plants, including *Arabidopsis*, rice, maize, brachypodium, sorghum, foxtail millet, barley, *Aegilops tauschii* and *Medicago truncatula* [7–11], but not yet in woody perennial fruit crops.

It is well known that CCT family members play diverse and important roles in flowering, circadian rhythms, development and abiotic stress tolerance [12]. The COL subfamily has been extensively studied and can be further subdivided into three smaller groups according to the degree of conservation and number of B-box domains [13–15]. In addition to the CCT domain, COL proteins contain one or two zinc-finger B-box domains towards the amino terminus, which are thought to be involved in protein–protein interactions [16, 17]. The first cloned CCT family gene in *Arabidopsis* was *AtCO*, which is required to promote photoperiodic flowering, at least in part by activating the expression of the *AtFT* (*flower time*) and *AtSOC1* (*suppressor of overexpression of CO1*) genes [18–20]. Other CO homlogues (*COLs*) have also been associated with abiotic stress tolerance, plant growth, development and metabolic processes, apart from playing a key role in photoperiodic flowering induction [21–26]. PRR subfamily genes contain a pseudoreceiver domain towards the amino terminus, as well as a CCT domain at the carboxy terminus [26]. PRRs are key genetic components of interconnected transcriptional-translational feedback loops that regulate circadian clock-output pathways [26–29]. In *Arabidopsis*, all *PRR* genes (*AtPRR1*, *AtPRR3*, *AtPRR5*, *AtPRR7*, and *AtPRR9*) that have redundant functions directly regulate the expression of genes implicated in abiotic responses, cell elongation, and photoperiodic flowering responses [26–28, 30]. In addition to the CCT domain, the ZIM subfamily contains both a C2C2-GATA zinc-finger domain and a TIFY domain [31, 32]. ZIM subfamily genes, including *ZIM* and *ZIM-like* (*ZML*) genes, are involved in hypocotyls and petiole elongation, cryptochrome 1-dependent responses to excess light and wound-induced lignification [33–35]. TCR1 and ASML2 subfamilies represent two distinct classes of the CCT family, and both encode proteins possessing just a single CCT domain [6, 7]. The *AtTCR1* gene has been shown to be transcriptionally induced by *Arabidopsis* endoplasmic reticulum stress [7]. *AtCIA2*, *AtCIL*, and their barley homologues (*HvCMF3* and *HvCMF7*) belong to the TCR1 subfamily and play critical roles in chloroplast development, thylakoid morphology, photosynthetic activity and various abiotic stress responses [36–39]. The functions of *ASML2* genes are generally not well understood. Overexpression of *AtASML2* results in enhanced expression of a subset of sugar-inducible genes in *Arabidopsis* [6].

As sessile organisms, plants need to depend on their ability to adapt to complex changes in the surrounding environment [40, 41]. As a consequence, they are equipped with sophisticated mechanisms that integrate environmental cues, such as light signalling (light quality, light intensity and photoperiod), and their endogenous regulators to optimize their growth and productivity [30, 42, 43]. There is increasing evidence that *CCT* genes are associated with light responses and/or photosynthetic capacity. *Ghd7*, the homologue of the *CCT* gene in rice, is a major locus that is responsible for natural variation in chlorophyll content at the heading stage [44]. Another *CCT* gene in rice, *CRCT* (CO_2_ responsive CCT protein), controls the capacity for starch synthesis, which can indirectly affect the photosynthetic rate under elevated CO_2_ conditions [45, 46]. In *Arabidopsis*, *AtBBX4* is a CCT domain protein with an abundance that is positively modulated by phyB under red light, thereby promoting photomorphogenic development [47].

Pear is one of the most economically important fruit crops in the world. In the field, the canopy architecture of pear is critical in determining the light environment and thereby indirectly affects the source–sink relationship [48]. To understand the potential mechanism of pear photosynthetic variability responses to heterogeneous light environments within canopies, we conducted physiological and transcriptomic surveys to capture progressive stages of photosynthetic differentiation between distinct canopy structures [49]. We found that the two orthologues (LOC103943360 and LOC103951583) of *AtPRR5* in pear were hub genes of the module positively correlated with pear photosynthetic rate and might play key roles in photosynthetic performance under distinct canopy microclimates [49]. Here, we address the important question of whether other members (*PbCCTs*) of the CCT family in pear could also participate in the regulation of photosynthesis and light signal response processes. However, little information is available on the identification and functional characterization of *PbCCTs* in pear, an important fruit crop. With the complete pear genome sequences of *Pyrus bretschneideri* publicly available [50], it is now possible to perform a genome-wide comprehensive analysis of *PbCCTs*. In this study, we identified *PbCCTs* and analysed the chromosomal locations, phylogenetic relationships and gene structure, as well as the expression patterns of some members in various tissues and in response to different light signals. In addition, transient expression analysis was used to investigate the possible roles of the *PbPRR2* gene, a key *CCT* member, in response to light signals. This comprehensive study of the CCT family may provide valuable information for further research and utilization of *PbCCTs*, helping to enhance our understanding of the possible roles of *PbCCTs* in the adaptation of pear to changing ambient light signalling.

## Methods

### Genome-wide identification of CCT family genes

The hidden Markov model profile of the CCT domain (PF06203) downloaded from the Pfam database (http://pfam.xfam.org/) was used for identification of the *PbCCTs* from the downloaded database of the Pear Genome Project (http://peargenome.njau.edu.cn/) using the HMMER programme (version 3.1b2) with a threshold e-value < 10e^−10^ [50]. Using the same criterion, CCT family sequences from *Arabidopsis thaliana* (ftp://ftp.ensemblgenomes.org/pub/plants/release-38/fasta/arabidopsis_thaliana/), *Oryza sativa* (ftp://ftp.ensemblgenomes.org/pub/plants/release-38/fasta/oryza_sativa/), and two other Rosaceae species (GDR; https://www.rosaceae.org/), including apple and woodland strawberry (*Fragaria vesca*, diploid wild species), were obtained. To confirm the reliability of the search results, the obtained sequences were further examined based on the presence of conserved domains of CCT proteins using the InterProScan software package (version 5.25–64.0). Finally, a self-blast of protein sequences was performed to remove redundancy. Alternative splice variants were not considered. Any two protein sequences that showed a perfect match were deemed to be redundant gene pairs, and the shorter sequence was removed from the potential *CCT* gene list.

### Analysis of synteny relationships, chromosomal locations, protein properties, gene structure and conserved motifs

The Multiple Collinearity Scan toolkit (MCScan X) was used to identify whole-genome duplication (WGD)/segmental and tandem duplications in the pear genome [51]. The KaKs_Calculator 2.0 was used to determine nonsynonymous (Ka) and synonymous (Ks) substitutions [52]. To exhibit the syntenic relationship of the orthologous *CCT* genes between pear and other selected species (apple*/*strawberry*/Arabidopsis*/rice), syntenic analysis was performed using MCScan X software. The physical location information for each *CCT* gene was retrieved from the pear/apple/strawberry genome database. The synteny relationships and location data among the three Rosaceae species (pear, apple and strawberry) were then plotted using TBtools software [53].

The molecular weights and theoretical isoelectric points of the PbCCT proteins were predicted by the compute pI/Mw tool in the ExPASY server (https://web.expasy.org/compute_pi/). The exon–intron structures of the *PbCCTs* were identified with the Gene Structure Display Server (GSDS, http://gsds.cbi.pku.edu.cn/) programme by the alignment of cDNA sequences with the corresponding genomic sequences. The InterProScan programme (http://www.ebi.ac.uk/interpro/) was used to characterize the domains and motifs of the pear CCT family.

### Phylogenetic analysis

To investigate the phylogenetic relationship between pear and *Arabidopsis*, unrooted neighbour-joining (NJ) trees were constructed using MEGA7 software with 1000 bootstrap replicates [54]. The numbers generated for each clade represent the bootstrap support values expressed as percentages. The same method was adopted to construct the NJ phylogenetic tree for the five subfamilies of the pear CCT family.

### Plant materials, growth conditions and treatments

To investigate the effects of light quality on *PbCCT* gene expression, one-year-old grafted seedlings of ‘Wonhwang’ (*P. pyrifolia* Nakai cv. ‘Wonhwang’) pear cultivar were obtained from the experimental orchard (30.292°N, 114.143°E) of the Research Institute of Fruit and Tea. Uniform and healthy plants were transplanted into plastic pots and cultured in an intelligent growth chamber (RLD-1000E-4, Le Electronic Instrument Co., Ltd., Ningbo, China) maintained at 25 °C and 70% relative humidity (RH). The commercially available light source used in this study was cool-red (R)/blue (B)/green (G) light-emitting diode (LED) panels. The distance between the lamps and pear leaves was ~ 20 cm. For the R light gradient treatments, fixed B light (1800 lx) and G light (5000 lx) intensities were also provided. The gradual increase in R light included six light intensity treatments, i.e., 500 lx (R500), 1000 lx (R1000), 1500 lx (R1500), 2000 lx (R2000), 2500 lx (R2500) and 3000 lx (R3000). For the B light gradient treatments, the seedlings were sequentially incubated in six B light intensity gradients, i.e., 1000 lx (B1000), 1500 lx (B1500), 2000 lx (B2000), 2500 lx (B2500), 3000 lx (B3000) and 3500 lx (B3500), while R light (2000 lx) and G light (5000 lx) were fixed light intensities. The fully expanded leaves from three individual plants were defined as three biological replicates. After light irradiation, leaf samples were harvested by rapid freezing in liquid nitrogen and stored at -80 °C until further use.

To test whether *PbPRR2* could regulate photosynthetic performance in response to varying R light signals, *PbPRR2* was transiently overexpressed in *N. benthamiana* leaves. One day after infiltration, *N. benthamiana* plants started to receive LED light treatment. The distance between the lamps and samples was ~ 30 cm. During the experiment, the temperature was maintained at 25 °C, while the RH was maintained at 70%. A gradient of R light was established, while B light (5000 lx) and G light (1800 lx) were relatively uniform. To determine the R light irradiance to which each leaf was exposed, light intensities (45–105 µmol m^−2^ s^−1^) were measured on the upper surface of each individual leaf using an LI-180 spectrometer (LI-COR Inc., USA).

### Measurements of the light environment among different pear tree canopy positions

To investigate the light environment among different pear tree canopy positions, sunlight spectra were measured with an LI-180 spectrometer at 5 cm above the surface of the leaves in a specific location. Adult ‘Wonhwang’ pear trees (ten years old) were grown in the experimental orchard of the Research Institute of Fruit and Tea. The experiment was carried out in a randomized complete block design with three replications. Trees from each block were randomly selected, which represented biological replicates. Each tested tree was divided into four canopy positions, i.e., sunny side-interior part of the canopy, sunny side-exterior part of the canopy, shady side-interior part of the canopy, and shady side-exterior part of the canopy. The interior and exterior parts of the canopy were approximately 0–1.0 m and more than 1.0 m away from the trunk, respectively. For each biological replicate of each canopy position, light quality parameters were measured at three independent positions (three technical replicates). All sunlight spectra measurements were performed every 2 h from 08:00 to 16:00 on sunny and clear days (105 days after flowering). All data were analysed by one-way ANOVA (IBM SPSS Statistics 19 software) followed by Duncan’s multiple range tests with a significance level of *P* < 0.01.

### Expression analysis of *PbCCTs*

To investigate the expression patterns of *PbCCTs* in different pear tissues, the normalized RPKM (reads per kilobase per million mapped reads) values of pear *CCT* genes were extracted from the previously published RNA-Seq data of leaf, ovary, petal, shoot, stigma, and fruit (15 days after full bloom) [55]. The results were visualized using a heatmap with transformed log_2_ (RPKM + 1) values using the ‘pheatmap’ R package (https://cran.r-project.org/web/packages/pheatmap/index.html).

To investigate the possible functions of the *PbCCTs* in varying light quality environments, *PbCCTs* with higher expression levels (RPKM values > 10) in leaves were selected for further analysis using qRT–PCR. Total RNA was extracted from the frozen leaves using the RNAprep Pure Plant Kit (Polysaccharides & Polyphenolics-rich) (Tiangen, China) according to the manufacturer’s protocol, followed by RNA integrity examination on 1.0% agarose gels stained with ethidium bromide. First-strand cDNA synthesis and qRT–PCR were performed as described previously [48]. Primer sequences for qRT–PCR analysis were designed using Primer Premier 5 (Additional file 1). Two reference genes, i.e., *PbSKD1* and *PbYLS8*, which were shown to be stably expressed in pear leaves [48], were used as internal controls to normalize the qRT–PCR data. Relative quantification was calculated according to the Ct method (2^−△△*C*T^). For each sample, three independent biological replicates were performed to acquire reliable results. Statistical analyses were performed as described above.

### Gene cloning, vector construction and transient transformation

The cDNA sequence of *PbPRR2* was amplified from ‘Wonhwang’ pear leaf cDNA using gene-specific primers (Additional file 1) and then cloned into the pDONR221 vector by the BP reaction (Gateway, Invitrogen). Several independent clones were sequenced to confirm the correct sequence of *PbPRR2* and then transferred by Gateway LR reaction into the destination vector pHEX2. All Gateway reactions were performed as recommended by the manufacturer (Thermo Fisher Scientific, https://www.thermofisher.com). Sequence alignment was performed using the Clustal X2 programme (http://www.clustal.org/) and GENEDOC. pHEX-*GUS*, pHEX-*PbPRR2* and pBIN61-p19 (suppressor of gene silencing p19, which could dramatically enhance transient expression of a broad range of proteins) constructs were separately introduced into *Agrobacterium tumefaciens* strain GV3101 by the electroporation method [56]. *A. tumefaciens* cultures carrying each expression vector were mixed with an equal volume of *A. tumefaciens* strain containing pBIN61-p19 and coinfiltrated into *N. benthamiana* leaves as described by Hellens et al*.* (2005) [57]. Three days after infiltration, photosynthetic measurements were taken for 20 leaves per construct (*n* = 20) under different R light levels using the portable CIRAS-3 photosynthesis system (PP. Systems Inc., USA). Data are expressed as the mean ± SEM and were analysed using IBM SPSS Statistics 19 software. The differences between means were determined by the Student’s *t test* with a significance level of *P* < 0.001.

## Results

### Identification and genomic distribution of the CCT family in pear

In total, 42 *PbCCTs* were identified in the pear genome (Additional file 2). CCT family members were classified into COL, PRR, ZIM, TCR1 and ASML2 subfamilies and then systematically named according to their family name and sequence similarity. Our analysis revealed that the COL subfamily consisted of the highest number of *CCT* genes in pear, with 33.3% (14 *PbCOLs*) of the total *PbCCTs* (Additional file 2). Both PRRs and ASMLs constituted the second largest subfamily, with 19.0% (8 *PbPRR*s and 8 *PbASML2s*) of the *PbCCTs*. The ZIM and TCR1 subfamilies were the smallest, with 14.3% (6 *PbZIMs* and 6 *PbTCR1s*) of the *PbCCTs*. The molecular weight of these PbCCT proteins ranged from 22.8 kD to 93.6 kD, and their isoelectric point values were between 4.27 and 9.45.

**Fig. 1:**
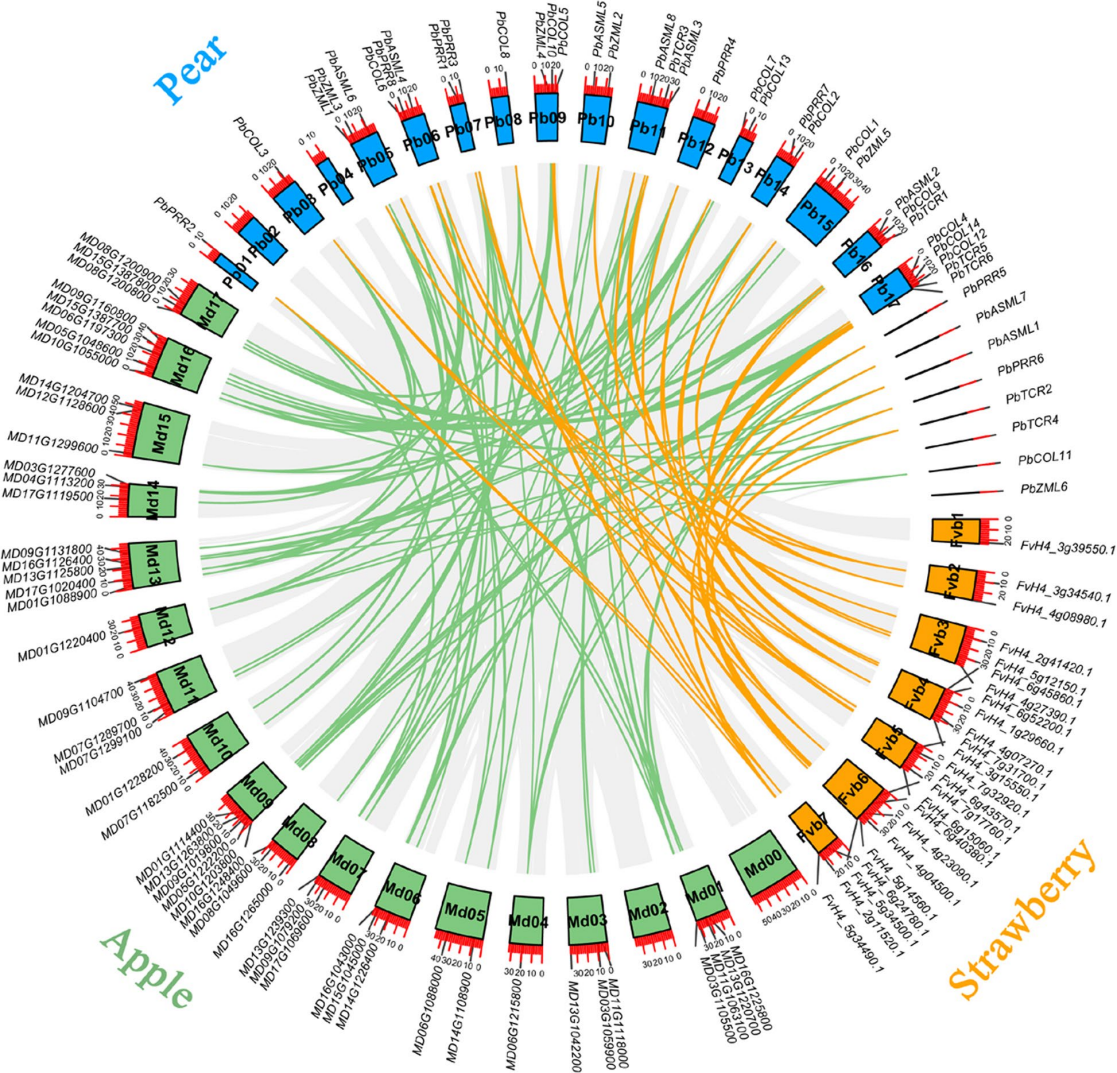
Localization and synteny of CCT genes in pear and two other Rosacease species. Chromosomes of pear, apple and strawberry are shown in sky-blue, dark sea-green and goldenrod colours. The approximate positions of each *CCT* gene are marked with short grey lines on the circles. Gene pairs with syntenic relationships are connected by coloured lines

*PbCCTs* were unevenly distributed over 15 of the 17 pear chromosomes, with no *PbCCT* gene found on chromosomes 2 and 4 (Fig. 1). Among these, chromosomes 1, 3, 8 and 12 contained the fewest *PbCCTs,* with only one member (2.4%) on each chromosome, while chromosome 17 possessed the highest number of *PbCCTs,* with five (11.9%) of the 42 members. However, it should be noted that eight *PbCCTs* remained on unmapped scaffolds. Similar to that in *PbCCTs*, the putative *CCT* genes in the other two Rosaceae species (apple and strawberry) also exhibited random chromosomal distribution (Fig. 1 and Additional file 3).

To clarify the potential mechanism of evolution of the *PbCCT* gene family, both WGD/segmental duplication and tandem duplication events were investigated in this study. Among all identified *PbCCTs*, a total of seven gene pairs were localized to WGD/segmentally duplicated regions, while there was no gene in tandem repeats (Additional file 4), indicating that WGD/segmental duplication events were the major contributors to the expansion of the pear CCT family. All members of the pear genome have undergone two genome duplication events, ancient WGD (Ks ~ 1.50–1.80) and recent WGD (Ks ~ 0.15–0.30) [50, 58]. The Ks values of two duplicated gene pairs were 0.162 and 0.207, implying that they might be derived from the relatively recent WGD event (approximately 30–45 MYA; MYA: million years ago); five duplicated gene pairs had smaller Ks values (0.004–0.041), suggesting that they might come from more recent segmental duplication events (Additional file 4). Moreover, with one exception (*PbTCR5*-*PbTCR6*), the Ka/Ks ratios of the other duplicated pairs were less than 0.26, implying that the pear *CCT* gene family had mainly undergone strong purifying selection (Additional file 4).

To further explore the synteny relationships of CCT family genes between pear (Rosaceae/Maloideae) and the other four representative species, *Arabidopsis* (dicot model plant), rice (monocot model plant), apple (Rosaceae/Maloideae) and strawberry (Rosaceae/Rosoideae), we performed interspecies comparative synteny analysis in a pairwise manner (Additional file 5). A total of 41, 33, 35 and 12 *PbCCT* genes were found to exhibit synteny relationships with *CCT* genes from apple, strawberry, *Arabidopsis* and rice, respectively, for example, *PbASML1* (pear)—*MD13G1220700*/*MD16G1225800* (apple)—FvH4_4g04500.1 (strawberry)—At2g33350 (*Arabidopsis*)—Os10t0466500-01 (rice). These results provide insights that will assist in the prediction of the possible roles of *PbCCTs*.

### Phylogenetic analyses of *CCT* genes

**Fig. 2:**
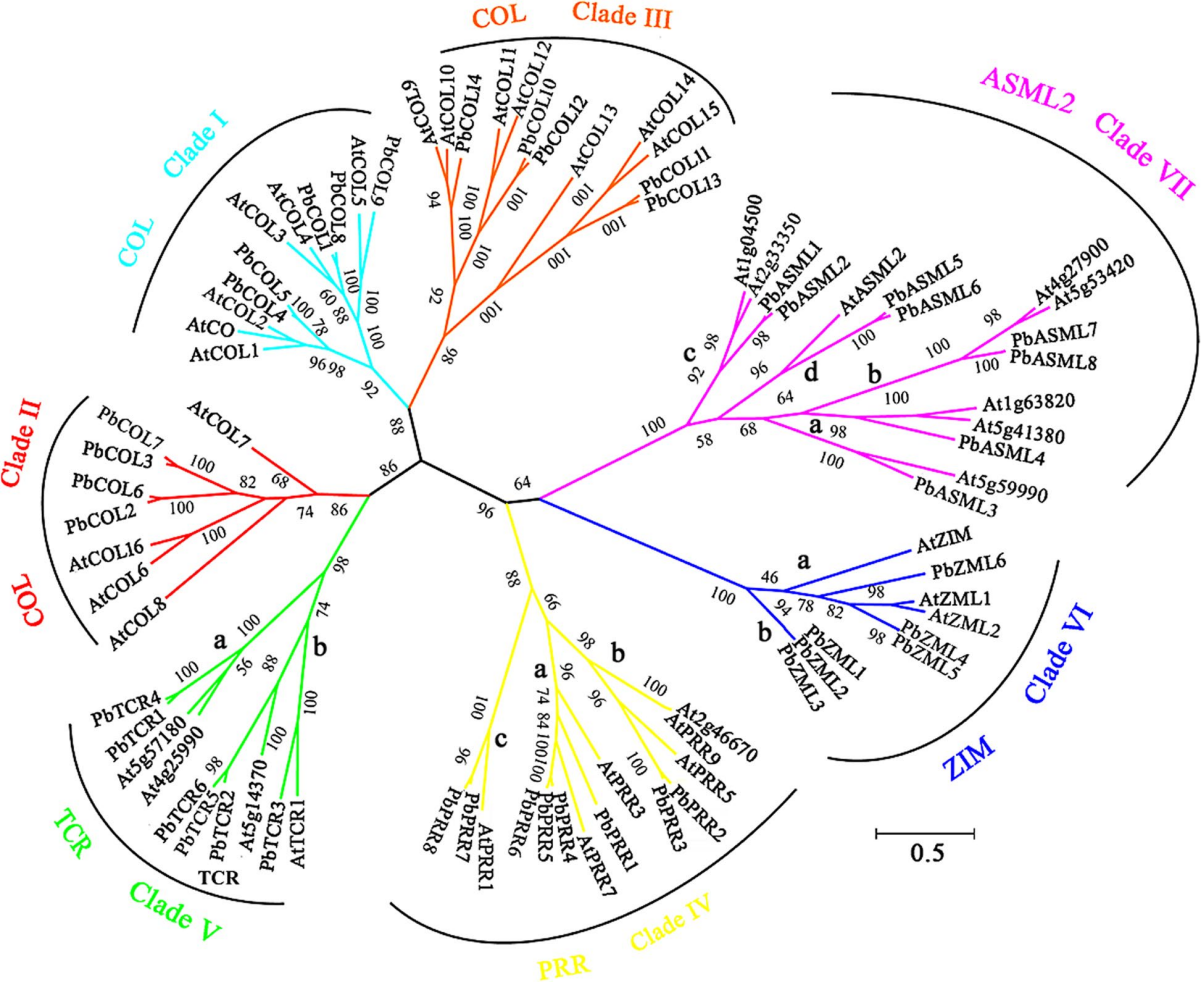
Phylogenetic relationships and subfamily designations in CCT proteins from pear and Arabidopsis based on the neighbour-joining method. The reliability of the predicted tree was tested by bootstrapping with 1000 replicates. The percentage of neighbour-joining bootstrap replications (> 40%) is shown above each node. These proteins were divided into seven clades and are represented by different colours

**Fig. 3:**
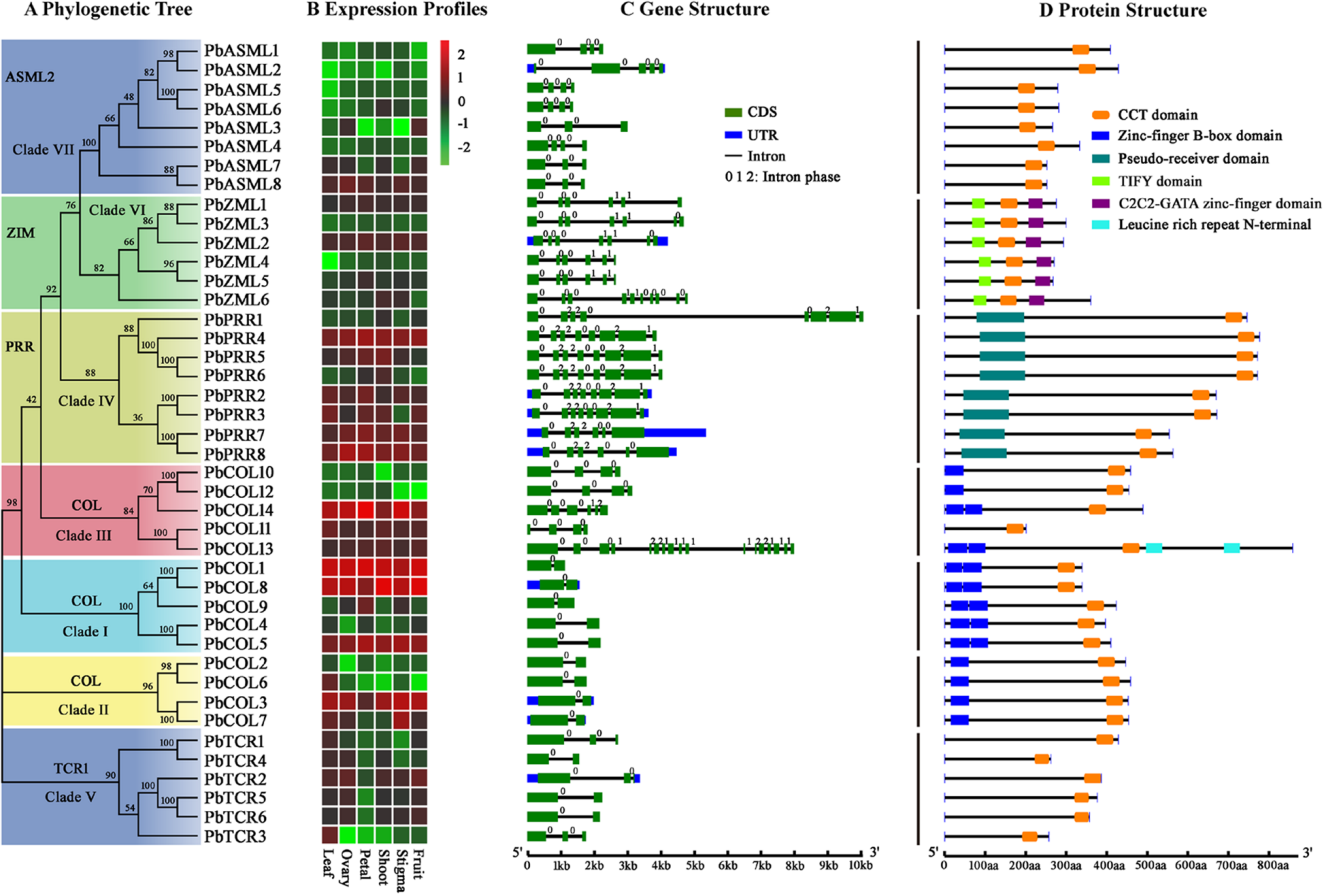
Phylogenetic relationships, expression profiles, gene structure and protein structure of pear CCT genes. A Neighbour-joining trees constructed for CCT genes from the ASML2, ZIM, PRR, COL and TCR1 families. **B** Heatmap showing the expression profiles of *CCT* genes in different tissues, including leaf, ovary, petal, shoot, stigma and fruit. The colour gradient from red to green indicates that expression values change from high to low. **C** Structure of *CCT* genes with exon(s) in green, UTR regions in blue, and solid lines between the coloured boxes indicating introns. The number indicates the phases of the corresponding introns. **D** Structures of CCT proteins with the CCT DNA binding domains represented by orange boxes, the zinc-finger B-box domain in royal blue, pseudoreceiver domain in aerugo, TIFY domain in green, C2C2-GATA zinc-finger domain in purple and leucine-rich repeat N-terminal domain represented by sky-blue boxes

To explore the phylogenetic relationship of the CCT family, an unrooted neighbour-joining phylogenetic tree was established based on the alignment of the full-length CCT protein sequences from pear and *Arabidopsis* (Fig. 2). In most clades, internal nodes were supported by confidence values of at least 80%, indicative of good consistency in the topology, which further corroborated the reliability of the tree. To test the reliability of the tree topology, protein domain architecture was used to provide additional support for the proposed phylogeny. The majority of members belonging to the same phylogenetic group exhibited common motif compositions (Fig. 3D). For example, TIFY and C2C2-GATA zinc-finger domains are specifically shared by the ZIM subfamily. The presence of the pseudoreceiver domain is also clade dependent in the PRR subfamily. The conserved intron/exon structural characteristics also supported the fine structure of the phylogenetic trees. For example, all the coding sequences of the *PbPRRs* were interrupted by 5 or 7 introns, while the TCR1 subfamily contained no more than two introns (Fig. 3C).

According to the classification criteria of the CCT family in *Arabidopsis*, *PbCCTs* were classified into seven major clades, Clades I-VII (Fig. 2). To clarify the phylogenetic relationships, Clades IV, V, VI, and VII were further divided into three, two, two, and four subgroups, respectively. Remarkably, we found that all seven clades included genes from both pear and *Arabidopsis*, indicating that ancestral genes of the seven clades diverged before the differentiation of pear and *Arabidopsis*. Interestingly, one subgroup, i.e., Clade VIb, included only *PbCCTs* and not *AtCCTs*, implying that these genes might have specialized roles that were either lost in *Arabidopsis* or were acquired after divergence from their common ancestor.

All *PbCOLs* were categorized into three clades, with well-supported bootstrap values: five *PbCOLs* in Clade I, four *PbCOLs* in Clade II, and five *PbCOLs* in Clade III (Figs. 2 and 3A). Clade I comprised five *PbCOLs* (*PbCOL1*/*4*/*5*/*8*/*9*), featuring a conserved CCT domain with two upstream zinc-finger B-box domains (Fig. 3D). *PbCOL* members (*PbCOL2/3/6/7*) in Clade II exhibited one B-box domain and a CCT domain. The gene structures of Clades I and II were highly conserved, containing two exons and one intron (Fig. 3C). The *Pb*COL homologues (*PbCOL10-PbCOL14*) were clustered with Clade III of the *Arabidopsis* COL subfamily, which possesses a normal B-box domain, a second divergent B-box domain and a CCT domain (Fig. 2). We found that this phylogenetic classification of *PbCOLs* in Clades I and II was the same as the classification of the *Arabidopsis* COL subfamily based on the difference in the B-box domain (Figs. 2 and 3D). However, *PbCOL10*, *PbCOL11* and *PbCOL12* in Clade III contained only one or no B-box domain (Fig. 3D). These patterns suggested that the corresponding genes might have lost the B-box-type zinc finger domain.

The PRR subfamily was further divided into three main subgroups based on their phylogenetic relationship, which were designated Clades IVa, IVb and IVc (Fig. 2). Four members (*PbPRR1*/*4*/*5*/*6*) of Clade IVa and two members (*PbPRR2*/*3*) in Clade IVb were highly conserved, containing eight exons and seven introns (Fig. 3C). Clade IVc included two pear PRR genes (*PbPRR7*/*8*) that clustered with the *AtPRR1* (*AtTOC1*) gene from the same branch (Fig. 2).

The TCR subfamily could be divided into two subgroups, i.e., Clade Va and Clade Vb (Fig. 2). For Clade Va, *PbTCR1* and *PbTCR4* were clustered with two *AtTCR* genes (At5g57180 and At4g25990). Clade Vb contained four pear members (*PbTCR2/3/5/6*), which were clustered with two *AtTCR* genes (*AtTCR1* and At5g14370).

All pear *ZIM* genes were also divided into two subgroups (Fig. 2). In detail, Clade VIa comprised three pear *PbZIM* members (*PbZML4*/*5/6*), which clustered with *Arabidopsis* ZIM subfamily genes (*AtZIM*, *AtZML1* and *AtZML2*), while three pear ZIM homologues (*PbZML1*/*2/3*) were identified as a distinct subgroup (Clade VIb) that had no counterpart in *Arabidopsis*.

Pear ASML2 members were classified into four subgroups and were characterized by only conserved CCT domains (Figs. 2 and 3D). Clades VIIa and VIIb were divided from the same branch and contained *PbASML3/4* and *PbASML7/8*, respectively. *PbASML1/2* were clustered to Clade VIIc, and *PbASML5/6* belonged to Clade VIId.

### Expression profiles of* CCT* genes in different tissues and under varying light signal environments

To investigate the tissue expression profiles of the *PbCCTs* in pear, we analysed their transcript levels based on publicly available RNA-seq data of different tissues, including leaf, ovary, petal, shoot, stigma and fruit (Fig. 3B). In general, the candidate *PbCCTs* showed variation in tissue expression patterns. Many *PbPRRs* and *PbCOLs* exhibited high transcript abundance levels in all six tissues, whereas most *PbASMLs* were expressed at relatively lower levels in multiple tissues. On the other hand, several *PbCCTs* exhibited tissue-specific expression. For example, *PbCOL6* and *PbTCR3* were mainly expressed in leaves, whereas *PbCOL9* showed the highest transcript abundance in petals. Some duplicated gene pairs also showed divergent transcript levels. For instance, *PbZML3* showed very low expression in six different tissues, whereas its duplicated gene, *PbZML2*, was highly expressed in all tested tissues.

**Fig. 4:**
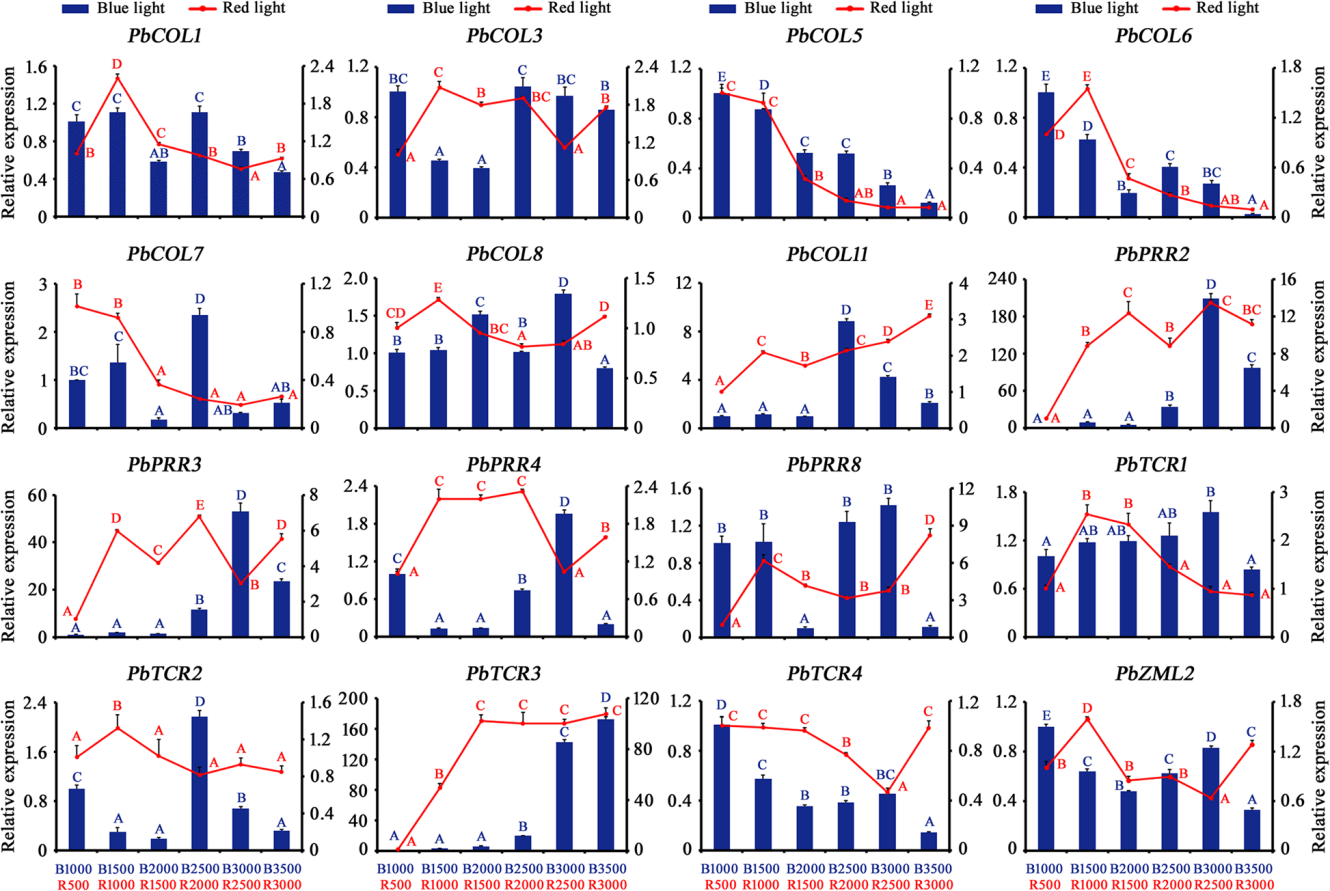
Expression profiles of 16 selected PbCCT genes under varying light signal environments. The Y-axes show the relative gene expression level under blue light treatment (blue bars, left) and the relative gene expression level under red light treatment (red line, right). B1000/1500/2000/2500/3000/3500 (x-axis) indicate six blue light intensity gradients, i.e., 1000 lx, 1500 lx, 2000 lx, 2500 lx, 3000 lx and 3500 lx. R500/1000/1500/2000/2500/3000 (x-axis) indicate six red light intensity gradients, i.e., 500 lx, 1000 lx, 1500 lx, 2000 lx, 2500 lx and 3000 lx. Error bars indicate the standard deviation from three biological replicates by qRT–PCR analysis. Different capital letters indicate significant differences at *P* < 0.01

We investigated the environmental light spectrum changes among different pear tree canopy positions. Compared with the exterior part of the canopy, the levels of R and B light decreased significantly in the interior part of the canopy, indicating that intensity changes in light quality were important signatures in fruit orchards (Additional file 6). Some genes from the *CCT* family have been shown to regulate growth and development by responding to R and B light signals [47, 59, 60]; therefore, the response of *PbCCT* induction to light quality treatments was further characterized by qRT–PCR. Overall, most of the selected *PbCCTs* showed highly diverse expression patterns under the enhancement of R/B light radiation (Fig. 4). These results suggested that they were sensitive to light quality signals, thus inducing different responses according to the external light conditions. Under the R light treatments, the expression of three genes (*PbCOL6*, *PbTCR1*, *PbTCR2*) reached a peak with low R light intensities (500 lx and 1000 lx) and was then downregulated during subsequent increased exposure to R light (Fig. 4). Additionally, *PbCOL11* and *PbTCR3* presented an increasing trend with increasing R light exposure. One homologous pair (*PbPPR2* and *PbPPR3*) displayed strong rhythmic expression patterns, suggesting that these genes could respond to R light changes during their regulation of pear growth and development. Notably, compared with R light at 500 lx (R500), the abundance of *PbPRR2* and *PbTCR3* transcripts dramatically increased more than 13.5-fold under a light intensity of 2500 lx (R2500) and 107.6-fold under a light intensity of 3000 lx (R3000), respectively. We also analysed the expression pattern of the *PbCCTs* in the B light treatment (Fig. 4). Among them, the transcript levels of four genes (*PbPPR2*, *PbPRR3*, *PbTCR1* and *PbTCR3*) were induced by enhanced B light, some of which decreased markedly at the highest abundance (B3500). Notably, the relative expression level of *PbPPR2* increased approximately 208.9-fold at an intensity of 3000 lx (B3000) relative to the control and that of *PbTCR3* increased approximately 172.0-fold at an intensity of 3500 lx (B3500). In addition, two *PbCCTs* (*PbCOL5* and *PbTCR4*) showed decreased expression levels in response to progressively increasing B light signals.

### *PbPRR2* might be involved in negatively regulating photosynthetic performance

Combining the previous transcriptomic study [49] and the present bioinformatics analysis and expression analysis, *PbPRR2* (LOC103943360), a close homologue of the *Arabidopsis* circadian clock gene *AtPRR5*, was chosen as a strong candidate for functional verification. Because *AtPRR5* is implicated in photomorphogenesis in R light, which is considered the most efficient wavelength for driving photosynthesis [61–64], these findings prompted us to investigate the potential role of *PbPRR2* under a broad range of R light intensities. *PbPRR2* has a 2013-bp open reading frame and encodes a protein of 670 amino acids (GenBank accession number: MZ826141). The amino acid sequences encoded by PbPRR2 and AtPRR5 (AT5G24470), the orthologue of PbPRR2 from Arabidopsis, were 40.12% identical (Additional file 7). The PbPRR2 protein featured a PR domain at the N-terminus and a CCT motif at the C-terminus.

**Fig. 5:**
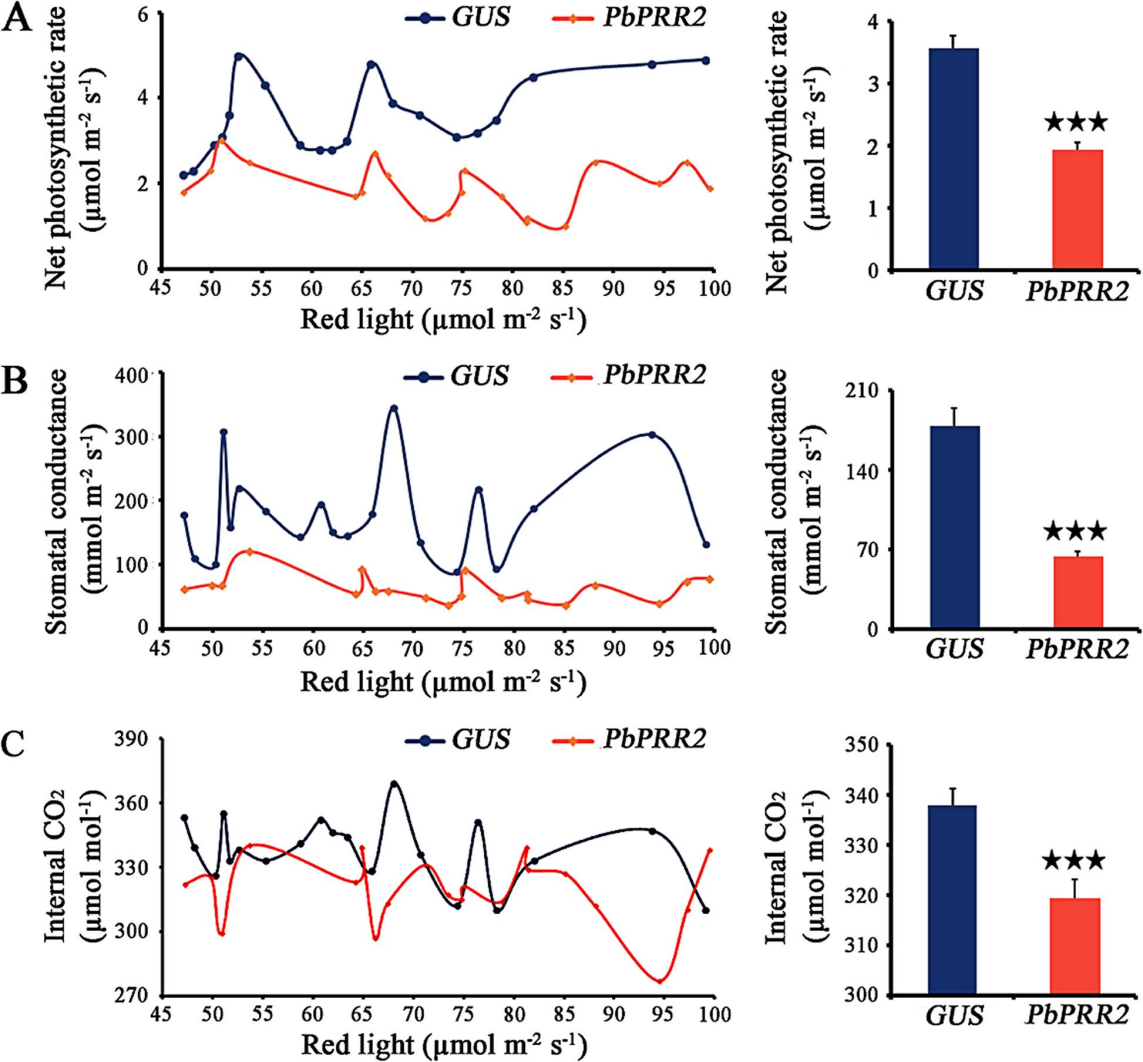
Photosynthetic performance of PbPRR2 transiently expressed in tobacco with increasing R light intensity. Leaves were infiltrated with *PbPRR2* (red line) or the control pHEX2-GUS (blue line). Data for three key photosynthetic parameters, i.e., net photosynthetic rate (**A**), stomatal conductance (**B**) and internal CO_2_ (**C**), are presented as the mean ± SEM (*n* = 20). Asterisks indicate significant difference at *P* < 0.001

To further test the possible role of *PbPRR2* in the regulation of photosynthetic properties under changing R light signals, *PbPRR2* was transiently overexpressed in *N. benthamiana* leaves and compared with the control leaves. Fluctuating profiles of net photosynthetic rate, stomatal conductance and internal CO_2_ were observed in both *PbPRR2*-infiltrated and control leaves with increasing R light intensity (Fig. 5). However, significantly reduced levels of these photosynthetic parameters were observed in leaves inoculated with the pHEX2-*PbPRR2* construct compared with the pHEX2-GUS control. These observations indicated that *PbPRR2* might suppress the red light-dependent enhancement of photosynthetic performance.

## Discussion

Photosynthetic performance is an important agricultural trait vital to the adaptation of horticultural crops to ambient light conditions [65]. Although *CCT* genes are well-established regulators of photoperiodic flowering pathways and circadian rhythms, their regulatory roles in other light-dependent agricultural traits, including photosynthesis, merit further exploration [10, 12, 61, 66]. Understanding the structural characteristics of pear CCT family genes and their specific responses to different light environments will help to identify some important candidates that may be involved in canopy light signal regulation. Here, we performed a comprehensive analysis of the PbCCT family genes. The number of *CCT* genes in pear (42) and apple (49) was nearly twice that in strawberry (24) (Fig. 1). In the process of genome evolution, gene duplication is always involved in gene family expansion, either through WGD, segmental duplication or tandem duplication [67, 68]. None of the *PbCCTs* were from tandem duplication, and all *PbCCT* gene duplications in pear were caused by WGD/segmental duplication events (Additional file 4); similar results were also observed for other gene families in pear [69–72]. The more recent WGD event presumably occurred in the ancestor of Maloideae, but not in Rosoideae [73, 74], which likely resulted in the higher gene numbers of the *CCT* gene family in pear and apple. After gene duplication, some duplicates may undergo functional divergence. The Ka/Ks ratios of the *PbCCT* duplication pair indicated that purifying selection was a major force driving the evolution of new functions for *PbCCTs*.

Phylogenetic analysis showed that most of the *CCT* family clades contained both pear and *Arabidopsis* proteins, suggesting that the two species displayed relatively conserved evolution (Fig. 2). Gene expression patterns of *PbCCTs* in response to varying light quality could provide important clues regarding gene functions during the light response process. For instance, two *PbCCTs* (*PbCOL5* and *PbCOL7*) showed progressively decreasing expression levels with increasing R light intensity (Fig. 4). *AtCO1*, an *Arabidopsis* orthologue of *PbCOL5* (Fig. 2), is a key player in the induction of flowering [18]. AtCO protein was degraded under R light by Phytochrome B [75]. Our results showed that shaded light had a lower intensity of R/B light (Additional file 6). These findings suggested that increased *PbCOL5* expression induced early flowering in response to the lower R light level to which pear shoots were exposed when shaded by neighbouring vegetation. Additionally, the expression of *AtCOL7*, which is a homologue gene of *PbCOL7* in *Arabidopsis* (Fig. 2), was rapidly downregulated in response to high R:FR (red:far-red light) [60]. Overexpression of *AtCOL7* was shown to enhance branching number under high R:FR conditions. It would be interesting to determine whether *PbCOL7* could increase branching by perceiving decreasing R light signalling.

In Arabidopsis, *AtCOL13* was shown to act as a positive regulator of R light-mediated inhibition of hypocotyl elongation [76]. The transcription level of *PbCOL11*, a homologue gene of *AtCOL13*, was upregulated robustly under the enhanced R light environment (Figs. 2 and 4), implying that *PbCOL14* might act in response to R light signalling to regulate photomorphogenesis in pear.

Our results provide evidence indicating that *PbPRR2* might have a novel role in the regulation of photosynthetic performance under varying light signal environments. Our phylogenetic analysis showed that the homologous pair (*PbPRR2/PbPRR3*) clustered together with Arabidopsis *AtPRR5* (Fig. 2). The functions of *AtPRR5* were implicated in the mechanisms underlying the control of flowering time and photomorphogenesis, as well as the circadian rhythm [61, 77]. Moreover, *AtPRR5-*ox plants were shown to be highly sensitive to continuous R light and seemed to be slightly hypersensitive to B light [77]. The “red or far-red light signalling pathway” was found to be an enriched category in *AtPRR5* direct targets [26]. Here, we noticed that the expression of *PbPRR2* and *PbPRR3* showed robust free-running rhythms under continuously increasing R light (Fig. 4). Our previous study indicated that these two genes, which are involved in the ‘circadian rhythm-plant’ pathway, were strongly correlated with photosynthetic performance [49]. A key piece of evidence supporting the role of *PbPRR2* in the light response was that it resulted in inhibition of photosynthetic performance compared with that of the control plants (Fig. 5). Therefore, *PbPRR2* (together with *PbPRR3*) should be taken into consideration for a better understanding of the molecular links between circadian rhythms and light signalling-controlled photosynthetic performance.

## Conclusions

In short, a total of 42 *PbCCTs* were identified in pear and divided into five subfamilies, as supported by phylogenetic relationships, intron–exon structures and conserved motifs. Expression analysis indicated that the PbCCT family might have diverse functions, and some members were sensitive to light induction, indicating that *PbCCT* genes were involved in light environmental adaptation. One *CCT* gene named *PbPRR2* was indicated to be associated with negatively regulating photosynthetic performance under the enhanced R light environment. All the results presented in this study provide comprehensive information on the CCT family in pear and lay a foundation for further research on the function of the *PbCCT* gene.

## Supplementary Information

**Additional file 1.** List of primers used inthis study.

**Additional file 2.** Information on *PbCCT* genes identified in the genome of pear.

**Additional file 3.** Lists of *CCT* genes identified in apple,strawberry, Arabidopsis and rice.

**Additional file 4.** Ka/Ks calculation of theduplicated *PbCCT *gene pairs in pear.

**Additional file 5.** One-to-one orthologousrelationships between pear and apple/strawberry/Arabidopsis/rice.

**Additional file 6.** Comparison of diurnalcourses of red light and blue light measured from different canopy positions. All measurements weretaken every 2 h between 08:00 and 16:00. Eachvalue represents the mean ± SEM (*n* = 9). The capital letters above the bars indicatesignificant differences (*P* < 0.01).

**Additional file 7.** Alignment of the amino acid sequences of PbPRR2 and AtPRR5. The redframe indicates the relatively conserved pseudoreceiver domain and CCT domain.

## Data Availability

All data generated or analyzed during this study are included in this published article and its supplementary information files.

## Abbreviations

Ka: Nonsynonymous substitution
Ks: Synonymous substitution
NJ: Neighbour-joining
RH: Relative humidity
R: Red
B: Blue
G: Green
LED: Light-emitting diode
RPKM: Reads per kilobase per million mapped reads
FR: Far-red
R500/1000/1500/2000/2500/3000: Six red light intensity gradients, i.e., 500 lx, 1000 lx, 1500 lx, 2000 lx, 2500 lx and 3000 lx
B1000/1500/2000/2500/3000/3500: Six blue light intensity gradients, i.e., 1000 lx, 1500 lx, 2000 lx, 2500 lx, 3000 lx and 3500 lx
WGD: Whole-genome duplication
MYA: Million years ago
