# Supplementary figures and images for: Genome-wide analysis of the CCT gene family in Chinese white pear (Pyrus bretschneideri Rehd.) and characterization of PbPRR2 in response to varying light signals
Source: BMC Plant Biol. 2022 Feb 23;22:81. doi: 10.1186/s12870-022-03476-1 (PMC8864873; doi:10.1186/s12870-022-03476-1)

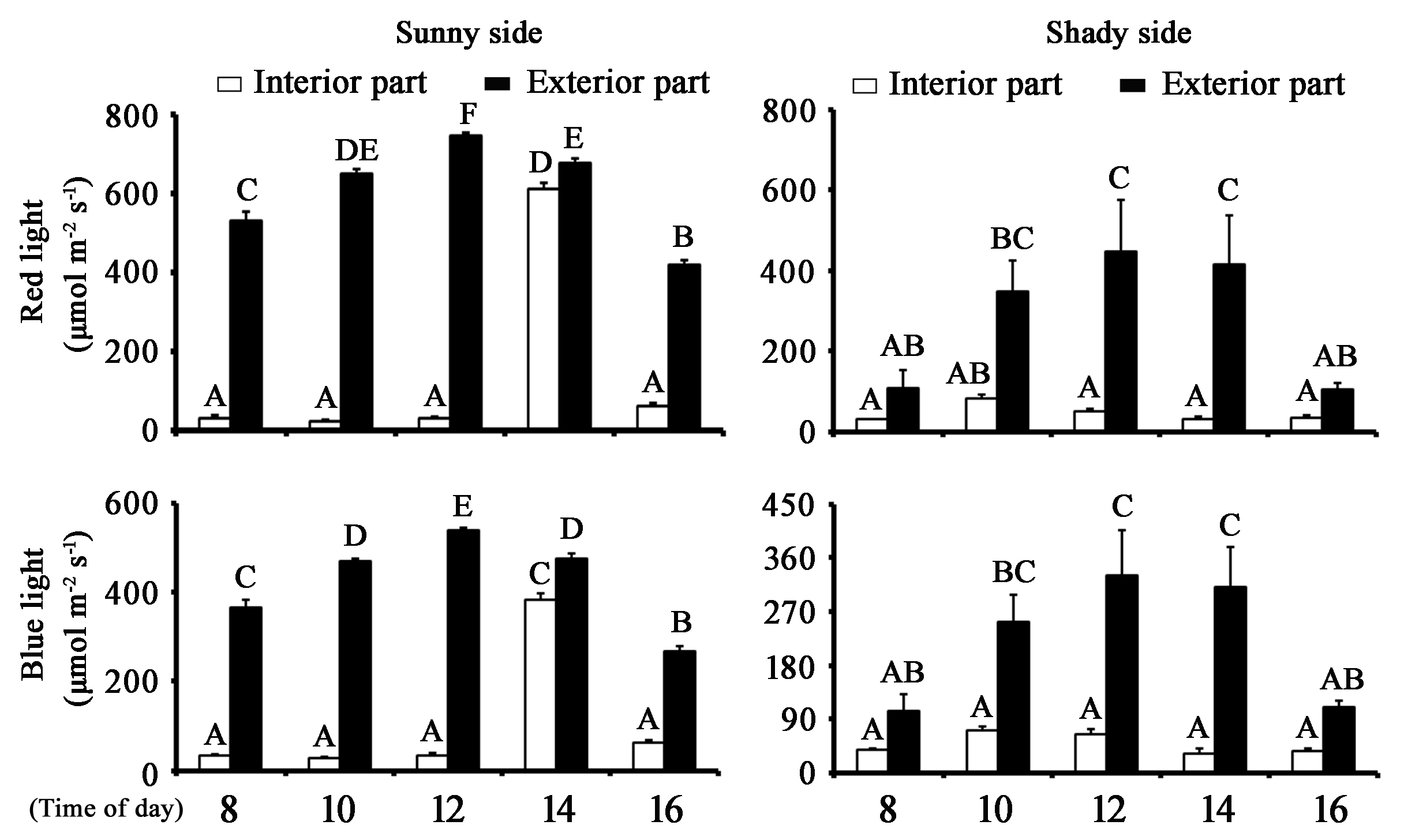

Supplement: Supplementary file 6 — Additional file 6. Comparison of diurnalcourses of red light and blue light measured from different canopy positions. All measurements weretaken every 2 h between 08:00 and 16:00. Eachvalue represents the mean ± SEM (n = 9). The capital letters above the bars indicatesignificant differences (P < 0.01). [file 12870_2022_3476_MOESM6_ESM.tif]

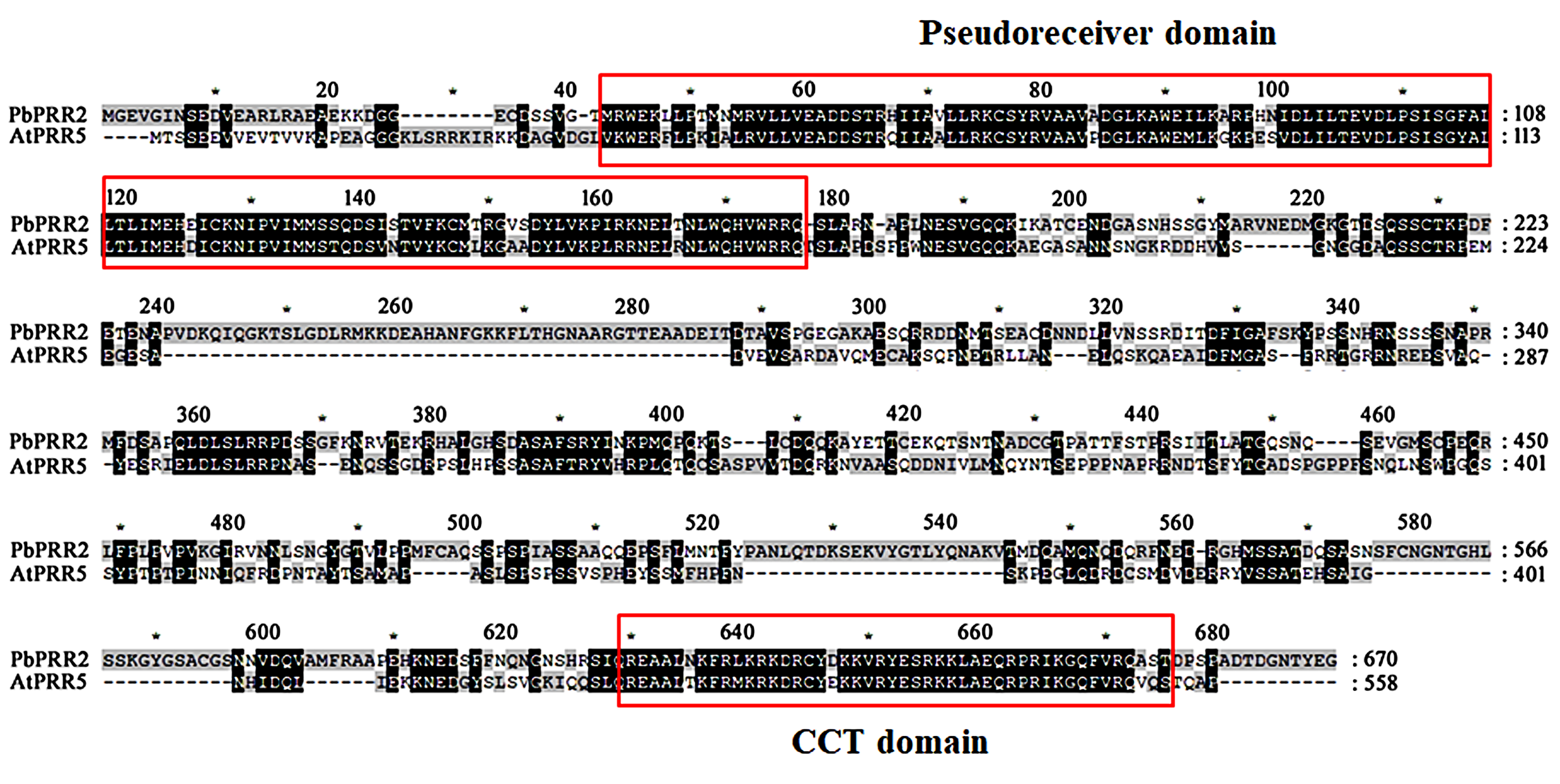

Supplement: Supplementary file 7 — Additional file 7. Alignment of the amino acid sequences of PbPRR2 and AtPRR5. The redframe indicates the relatively conserved pseudoreceiver domain and CCT domain. [file 12870_2022_3476_MOESM7_ESM.tif]
